# Perindopril Induces TSP-1 Expression in Hypertensive Patients with Endothelial Dysfunction in Chronic Treatment

**DOI:** 10.3390/ijms18020348

**Published:** 2017-02-07

**Authors:** Valentina Buda, Minodora Andor, Lucian Petrescu, Carmen Cristescu, Dana Emilia Baibata, Mirela Voicu, Melania Munteanu, Ioana Citu, Calin Muntean, Octavian Cretu, Mirela Cleopatra Tomescu

**Affiliations:** 1 Faculty of Pharmacy, Victor Babeş University of Medicine and Pharmacy, 2 EftimieMurgu, 300041 Timisoara, Romania; buda.valentina.oana@gmail.com (V.B.); mavoicu@yahoo.com (M.V.); 2 Faculty of Medicine, Victor Babeş University of Medicine and Pharmacy, 2 EftimieMurgu, 300041 Timisoara, Romania; andorminodora@gmail.com (M.A.); danaemilia@yahoo.com(D.E.B); citu.ioana@umft.ro (I.C.); calin.muntean@gmail.com (C.M); tavicretu@yahoo.com (O.C.); tomescu.mirela@umft.ro (M.C.T.); 3 Faculty of Pharmacy, VasileGoldis Western University, 86 LiviuRebreanu, 310045 Arad, Romania; anaionescuro@yahoo.com

**Keywords:** TSP-1, thrombospondin-1, endothelial dysfunction, essential arterial hypertension, perindopril, antihypertensive drugs, anti-inflammatory, anti-proliferative, angiotensin-converting enzyme (ACE) inhibitors

## Abstract

Thrombospondin-1 (TSP-1) is a potent endogenous inhibitor of both physiological and pathological angiogenesis, widely studied as a target in drug development for treating cancer. Several studies performed in the cardiovascular field on TSP-1 are contradictory, the role of TSP-1 in the physiopathology of cardiovascular disorders (CVDs) being, for the moment, incompletely understood and may be due to the presence of several domains in its structure which can stimulate many cellular receptors. It has been reported to inhibit NO-mediated signaling and to act on the angiogenesis, tissue perfusion, endothelial cell proliferation, and homeostasis, so we aimed to quantify the effect Perindopril has on TSP-1 plasma levels in hypertensive patients with endothelial dysfunction in comparison with other antihypertensive drugs, such as beta blockers, calcium channel blockers, and diuretics, in a chronic treatment. As a conclusion, patients under treatment with Perindopril had increased plasma levels of TSP-1 compared with other hypertensive patients and with the control group. The results of this study confirms the pleiotropic properties of Perindopril: anti-proliferative, anti-inflammatory, with effects showed by quantifying a single biomarker: TSP-1.

## 1. Introduction

Thrombospondin-1 (TSP-1) is a potent endogenous inhibitor of both physiological and pathological angiogenesis, widely studied as a target in the drug development for treating cancer. Recently, studies showed that it also inhibits NO-mediated signaling. Thus, it acts on angiogenesis, tissues perfusion, endothelial cell proliferation, and homeostasis [1,2,3,4].

TSP-1 is the most studied member of the family of thrombospondins, a family which consists of five multimeric, multidomain calcium-binding glycoproteins that act as regulators of cell-cell and cell-matrix associations, which interact with other extracellular matrix molecules that can influence their function [5].

A major source of TSP-1 is the alpha-granules of platelets which release this substance upon activation. It is also present in various cell types, such as endothelial cells, vascular smooth muscle cells, macrophages, and cardiomyocites [6,7,8,9,10]. It is involved in many processes that regulate cellular adhesion, migration, cell proliferation, apoptosis, vascular systemic tone, and cytosckeletal organization [11,12]. Being constitutively present within the blood vessels, it interacts with many important proteins that are involved in maintaining homeostasis and the vascular structure. This has been classified as a counter-adhesive protein that is capable of interacting with various cell-surface receptors, growth factors, bioactive molecules, proteases, and several studies have shown its implications in the pathogenesis of many cardiovascular diseases [2,13,14,15,16,17,18,19].

The studies performed in the cardiovascular field on thrombospondin-1 are contradictory and often conflicting, the role of TSP-1 in the physiopathology of cardiovascular disorders (CVDs) being far from completely understood. This controversy upon the effects of TSP-1 reported in various cell types may be due to the presence of several domains in its structure that can stimulate many cellular receptors [5,6,18,19].

TSP-1, a homotrimeric glycosylated protein of a 450 kDa, consists of N-terminal and C-terminal globular domains, connected by a thin strand. The N-terminal domain can be cleaved by several proteases, such as thrombin, trypsin, and plasmin; therefore, it can exist in a soluble state or associated with activated platelet membranes [19,20,21]. The C-terminal region allows it to form covalent bonds with other proteins (e.g., thrombin). The cellular receptors with which the thrombospondin-1 can interact include: CD36, CD47 (integrin associated protein), proteoglycans, and various integrins [6,22]. As we already mentioned it, the N-terminal domain present in the structure of TSP-1 has a heparin binding domain that can bind to heparin sulphate proteoglycans [23], the peptide sequence CSVTCG interacts with CD36 [24], and the C-terminal domain binds to CD47 [25]. TSP-1 can also bind to components of fibrinolytic system (e.g., plasminogen, an inhibitor of plasminogen activator inhibitor-1, urokinase), to cathepsin G, to various integrins (α3β1, αIIbβ3), and to elastase [26,27]. By binding to CD36 (the primary TSP-1 receptor, expressed on a variety of cells), TSP-1 induces anti-angiogenic and inflammatory actions, platelet aggregation, and adhesion to endothelial cells [20].

TSP-1 stimulation of CD47 (a member of immunoglobulin superfamily, component of the β3 integrin complex, expressed on T cells and polymorphonuclear cells, having a role in maintaining immune tolerance) induces the inhibition of releasing IL-2 [28,29]. The C-terminal domain of TSP-1 that is bound to CD47 mediates an anti-proliferative effect on vascular smooth muscle cells (VSMCs) [1].

TSP-1 can also stimulate and activate TGF (transforming growth factor)-β_1_, being the only member of its family capable to perform this action. Summarizing all of the information presented above, the role of TSP-1 in the cardiovascular disorders is far from being elucidated and understood and it probably depends on the exact physiopathological condition and on the context of its expression [5,6,19].

In this study, we aimed to quantify the variations of the TSP-1 plasma levels under different antihypertensive regimens, in hypertensive patients with endothelial dysfunction.

Having, as a starting point, the fact that Perindopril not only decreases hypertension, but it also possesses pleiotropic properties (due to the accumulation of bradykinin), by which it ameliorates the endothelial dysfunction, this study, therefore, compares its effect on TSP-1 plasma levels versus other antihypertensive regimens, as a chronic treatment, in patients with hypertension (HTN) and endothelial dysfunction (ED).

## 2. Results

Table 1 shows the clinical and demographic characteristics of all 351 patients included in this study. The two hypertensive groups (B and C) were similar in terms of age, blood pressure, and sex distribution, although the duration of hypertension was greater in Group B (patients treated with other antihypertensive medication, except Perindopril), without any statistical significance between the groups.

Table 2 shows the biochemical data for all of the groups. A significant difference between groups can be noticed in what concerns the cholesterol level of Group C (patients under treatment with Perindopril), due to a possible mild dislipidemia. Additionally, the creatinine levels were higher in this group, with mild renal impairment being one of the first choices for angiotensin-converting enzyme (ACE) inhibitors in hypertensive patients, as a result of their action of decreasing the proteinuria and offering renal protection due to the amelioration of ED.

Furthermore, it can be noticed that a higher level of systemic inflammation in Group C was statistically significant, reflected through the erythrocyte sedimentation rate (ESR) levels.

Concerning the other parameters, there were no significant statistical differences between the groups having the plasma values being in the normal range.

Table 3 shows the principal indicators of the presence/absence of ED in the three groups. The patients from Group A had no endothelial dysfunction (reflected throughout the flow mediated vasodilatation—FMD value), compared with the other two groups of hypertensive patients. In Group B, the degree of ED was higher, compared with Group C and statistically significant. The plasma levels of high-selectivity C reactive protein were higher in Group C, compared with the levels of other groups, and also the number of people who were smokers was higher in this group. The TSP-1 plasma level was also the highest in this group, being very close to the value considered statistical significant (*p* < 0.05). The intima-media thickness (IMT) values were greater in both groups of hypertensive patients. Additionally, the pentraxin-3 (PTX3) plasma levels were the smallest in the C group who were under treatment with perindopril.

The echocardiography showed a septal hypertrophy for all hypertensive patients (IVS > 11 mm) compared with the control group, without any statistical significance between the three groups. (Table 4). All of the other echocardiographic parameters were within their normal range, in all three groups, without any significant difference.

The analyses performed in order to determine the correlations between the plasma levels of TSP-1 and other parameters in both groups of hypertensive patients, and in the control group (Table 5, Table 6 and Table 7) showed that TSP-1 correlated positively (*r* > 0.3) only in Group B, with tryglicerides, leucocites, and neutrophils.

**Figure 1:**
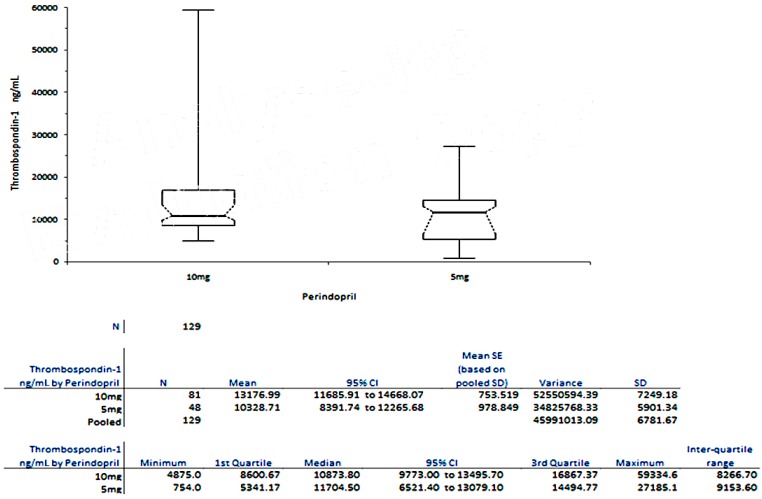
Variation of TSP-1 plasma levels in Group C, under different concentrations of Perindopril.

Figure 1 shows that patients treated with the lowest concentration of Perindopril (5 mg/day) had the lowest TSP-1 plasma levels, compared with those who were under treatment with the highest concentration of Perindopril available on the market (10 mg/day).

**Figure 2:**
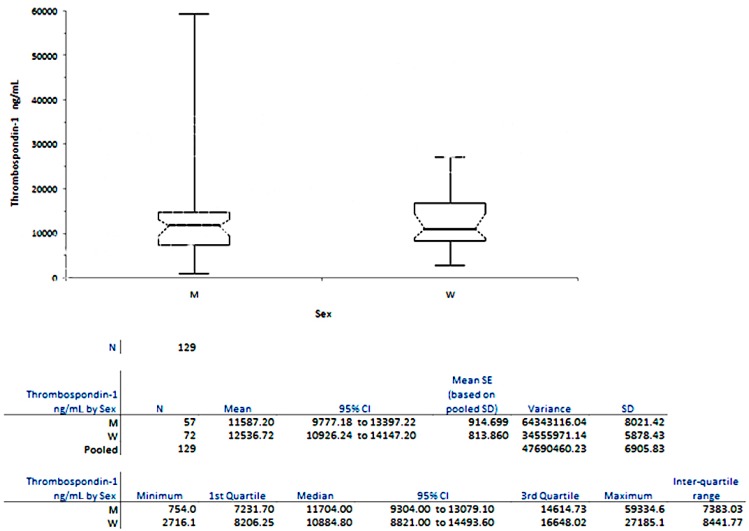
Variation of TSP-1 plasma levels in men and women in Group C.

From Figure 2 it can be noticed that women have higher plasma levels compared with men.

**Figure 3:**
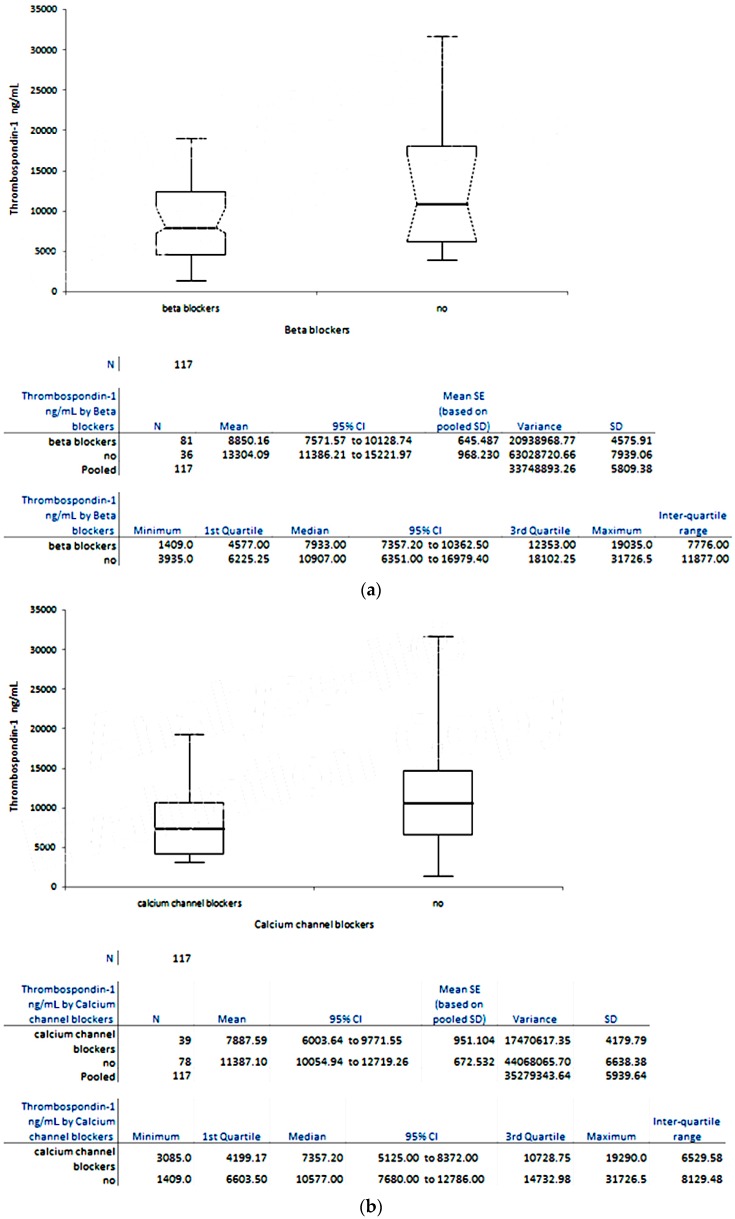

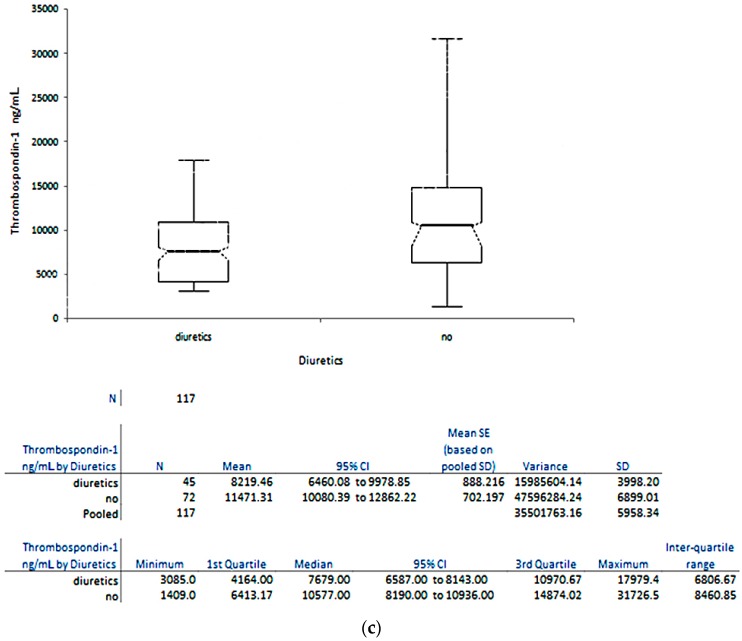
Variation of TSP-1 plasma levels in Group B, under different antihypertensive treatment: (**a**) beta blockers, (**b**) calcium channel blockers, and (**c**) diuretics.

From Figure 3 it can be noticed that patients under treatment with different classes of antihypertensive drugs (beta blockers, calcium channel blockers, and diuretics) had TSP-1 plasma levels lower than those under treatment with Perindopril (Group C).

## 3. Discussion

Perindopril belongs to the third generation of ACE inhibitors, intensively prescribed and studied. ACE inhibitors are a group of drugs used and listed as first-line agents in the treatment of hypertension, congestive heart failure, myocardial infarction, and left ventricular systolic dysfunction, and are either used alone or in combination with other types of drugs with different action mechanisms [30,31,32,33,34,35,36]. All of the positive effects on the pathologies mentioned previously are the consequences of their mechanism of action: firstly, they block the angiotensin (Ang)-converting enzyme the enzyme that transforms Ang I (found in the vascular endothelium of lungs and other organs and in lower concentrations in the vascular plasma) in Ang II, decreasing the circulating levels of Ang II. Due to the decreased synthesis of Ang II, the plasmatic levels of Ang I are increased, and through a feed-back phenomenon, Ang II induces the stimulation of the rennin synthesis and lowers the levels of angiotensinogen, due to its high consumption. The decreased levels of Ang II induce decreased effects of the peptide on the vascular tonus, the synthesis and liberation of aldosteron, the sodium-potassium balance, etc.

Secondly, due to the fact that ACE is identical with the enzyme that degrades bradykinin (BK), kinase II, Perindopril and other ACE inhibitors increase the bradykinin concentration and stimulate the action of endogenous kinins to produce nitric oxide (NO), prostaciclins, and EDHF (endothelium-derived hyperpolarizing factor), through the stimulation of bradykinin B_2_ receptors, having, as a consequence, the vascular protective effects and the reversed ED installed [34,36,37]. The inhibition of the BK degradation is the main mechanism (known at the moment) by which ACE inhibitors induce cardiovascular protection. This effect may differ between ACE inhibitors as it depends on the degree of the tissue affinity and on their binding to BK. Additionally, ACE inhibitors have a higher affinity for bradykinin sites compared with the affinity for Ang I, so they primarily inhibit BK degradation and then the production of Ang II [38].

We choose Perindopril for this study because of its pharmacokinetics and pharmacodynamic properties: the highest bioavailability (75%), the highest terminal elimination half-life of the major active ingredient (30–120 h), a higher time to reach maximum plasma concentration, the highest affinity for BK binding sites when compared with other substances from its class: Enalapril, Lisinopril, Quinapril, Ramipril [39], high lipophilicity and tissue penetration among other ACE inhibitors [38], prolonged duration of action [39], prolonged inhibition of ACE (>48 h) [40], and 24 h efficacy, which implies a low number of administrations per day and a higher compliance for patients to the treatment [39]. Moreover, it has been shown to exert potent anti-apoptotic actions on the endothelium and on the cardiac myocytes [38]. Regarding the study that we performed, the results presented in Table 2 and Table 3 clearly suggest the fact that, although the patients under chronic treatment with Perindopril have more risk factors compared with those under treatment with other antihypertensive drugs (beta blockers, calcium channel blockers, diuretics)—this conclusion being based on higher cholesterol levels, BMI, number of smokers, level of systemic inflammation (ESR and hs-CRP plasma levels)—the degree of ED is lower in this group (reflected by the FMD level) and the degree of local endothelial inflammation is also lower in the same group (reflected through the PTX3 plasma levels, an acute inflammatory marker, secreted by the endothelial cells). We can conclude that, based on these parameters, the endothelial function is better preserved by an ACE inhibitor than any other antihypertensive drugs (beta blockers, calcium channel blockers, diuretics) in a chronic treatment.

As for the TSP-1 plasma levels, although the difference between the groups have not reached the level of statistical significance (*p* = 0.08, *p* < 0.05), the plasma values of this biomarker are higher in the group of patients treated with Perindopril (Group C). Based on the evidence reported in the literature and discussed in the introduction about the effects of TSP-1 on the vascular endothelium (although they are contradictory and opposing), in our study, and based on the studies reported by others, the low levels of TSP-1 might be an inducer of VSMC proliferation and that higher levels of TSP-1 could induce an anti-proliferative effect on VSMCs by reducing its density (throughout the binding of TSP-1 C-terminal domain to CD47-binding peptide) [40,41,42,43]. Thus, in normal low levels, TSP-1 could act as an inducer of VSMC proliferation and, in pathological conditions (e.g., atherosclerosis), whereas TSP-1 is upregulated, high levels of TSP-1 could act as an anti-proliferative agent, although the effects of TSP-1 could be different in function of the pathology concerned [6].

TSP-1 has been also reported to play an important role in decreasing inflammation and, thus, having an anti-inflammatory action, due to its interaction with CD47 receptor expressed on T-cells and polymorphonuclear cells [44]. So, based on our study, we may say that, in Group C, the patients under chronic treatment with Perindopril, have a more decreased level of endothelial inflammation, reflected through the TSP-1 plasma levels and not only through PTX3.

No correlation had been found between TSP-1 serum levels and the disease severity and/or duration, as Table 5 and Table 6 show.

The data found in the literature about the relationship between TSP-1 and leucocytes [45,46,47,48] confirms the same idea found in our study, that TSP-1 plays an important role in the recruitment of monocytes and macrophages to the site of tissue inflammation or injury. In the present study, we found positive correlation between TSP-1 and triglycerides, leucocytes, and neutrophils, correlations that show the local, vascular, inflammatory site in patients under chronic treatment with other antihypertensive drugs, except Perindopril. This is one more argument that Perindopril controls better the local endothelial inflammation and the vascular function.

In a study performed by Xia et al. in 2011 [49], authors suggested that TSP-1 expression might be involved in the regulation of fibroblast phenotypes and that it could prevent the left ventricular hypertrophy in pressure-overloaded hearts, so we may add another positive effect proved by this study for Perindopril: its anti-fibrotic effect.

We also found that the TSP-1 plasma levels are lower for men than for women.

This study has several limitations, including the relatively low number of patients examined and the short observation time. Additionally, the study results cannot explain the molecular mechanisms by which Perindopril increases plasma TSP-1 levels in hypertensive patients with endothelial dysfunction; therefore, additional studies are needed to fully assess the effects of TSP-1 on vasculature.

## 4. Materials and Methods

This prospective, comparative study was performed in the Cardiology Clinic of the City Hospital of Timisoara (a city in the western part of Romania), from February 2015 to July 2016. It involved a number of 351 patients, 105 normotensive patients (Group A), and 246 hypertensive patients (Group B and C), matched by age and sex.

### 4.1. Patient Selection

The normotensive patients (Group A), the control group, were initially examined for suspicion of HTN, but the diagnosis had not been confirmed by the 24 h ambulatory monitoring device.

The hypertensive patients were divided into two subgroups: Group B (*n* = 117) included patients under chronic treatment with a β blocker (nebivolol 5 mg/day; metoprolol 50 or 100 mg/day; or bisoprolol, 5 or 10mg/day), a calcium channel blocker (amlodipine, 5 or 10mg/day; or lercanidipine, 10 or 20 mg/day), or a diuretic (indapamide, 1.5 or 2.5 mg/day; or furosemide, 20 mg/day + spironolactone, 50 mg/day).

Patients in Group C (*n* = 129) were under chronic treatment with Perindopril (5 or 10 mg/day), an angiotensin-converting enzyme (ACE) inhibitor.

Hypertensive patients were included under the following criteria: age >18 years old, who were diagnosed with essential arterial hypertension (blood pressure levels >140/90 mmHg) for at least one year and had received monotherapy with one of the drugs mentioned above.

Patients with other pathologies including atherosclerotic disease, diabetes, coronary artery disease, heart failure, kidney disease, asthma, hepatic disease, and acute or chronic inflammatory conditions were excluded. Additionally, patients treated with other ACE inhibitors or ARBs (antagonist receptor blockers) were excluded from this study due to the similar action in reversing the endothelial dysfunction.

All of the participants from the present study agreed to participate voluntarily and provided written informed consent.

Hypertensive patients (Group B and C) underwent screening including a physical examination and a medical history (duration of hypertension, current treatment, familial history, and other associated medical pathologies).

### 4.2. Laboratory Analysis

Patients were fasted for >10 h and venous blood samples were withdrawn early in the morning, in a temperature-controlled room. Standard biochemical analysis (e.g., serum glucose, creatinine, triglycerides, total cholesterol) was performed in the hospital laboratory by routine methods. Plasma concentrations of hs-CRP (high-sensitivity C-reactive protein), PTX3 (pentraxin-3), and TSP-1 were determined at Bioclinica SA Laboratory in Timisoara. PTX3 and TSP-1 plasma levels were determined by quantitative sandwich enzyme immunoassays (R and D Systems, Minneapolis, MN, USA) and plasma hs-CRP levels were measured using CRP Ultra Kits (Abbott Diagnostics, Wiesbaden, Hesse, Germany), using a highly sensitive immunoturbidimetric method. All of these methods are standardized methods, so we consider that there is no need to repeat the methods here.

### 4.3. Arterial Pressure

Blood pressure was measured three times in the right brachial artery of each patient, in the same temperature-controlled room, after 30 min of rest, with the patient in a supine position. The reported value of pressure was expressed as the mean of three measurements. We calculated the mean arterial blood pressure as follows: (2 × diastolic pressure + systolic pressure)/3.

### 4.4. Assessment of Flow-Mediated Dilation (FMD)

The assessment of flow-mediated dilation is, nowadays, the most common, non-invasive technique and is used to evaluate the vascular endothelial function in humans. It was developed in 1992 and, since its inception, ongoing efforts have been made to perform the original methodology in a more accurate, exact, and precise method. It consists of the capacity of the blood vessel to adapt to the increasing of blood pressure, a process that is dependent of the endothelium production of NO [50,51,52,53].

This procedure was performed after the patient fasted, stopped taking the vasoactive medication, and did not smoke for at least 10 h, based on the literature data. After resting for 10 min in a quiet room, the patient was placed in a supine position. His arm was placed in a comfortable position and the brachial artery was imaged above the antecubital fosa in the longitudinal plane, using a linear transducer in 9 MHz mode. The diameter of the brachial artery was measured manually with electronic calipers. In order to produce the local ischemia (to evaluate the ability of the endothelium to produce NO), the cuff of a manometer was inflated to a pressure of 50 mmHg higher than the systolic blood pressure of the patient. After 5 min of hyperemia, the cuff was released, increasing the diameter of the brachial artery. The maximum diameter was measured 1 min after the cuff release and the FMD was defined as the percent change in diameter from the rest to 1 min after the ischemia, considering a 20% increase as normal (healthy endothelium) and under 20% as endothelial dysfunction.

### 4.5. Carotid Intima-Media Thickness (IMT)

Carotid intima-media thickness was determined, as agreed in the Mannheim Consensus [54], at the common carotid artery at baseline in both carotid arteries, with the same equipment, a General Electric medical system VIVID S5 (General Electric Co., Horten, Norway) equipped with a 9 MHz linear array transducer (General Electric Co., Horten, Norway). Patients were examined in a supine position and the IMT was given by the built-in software (5432774-167, General Electric Co., Horten, Norway) of the ultrasound system. The reported value was the mean values of three measurements in each patient for the left and for the right carotid, 1 cm before the bifurcation. The following were considered as normal values: 0.9–1.1 mm, and values >1.2 mm as the presence of atherosclerotic plaque.

### 4.6. Echocardiography

The echocardiography was performed using a high-resolution ultrasonography medical system, VIVID S5, to assess the effects of hypertension on the structures and functions of the heart. We excluded patients with low ejection fractions (<40%), to avoid the interference in our results regarding the ED. The parameters evaluated included the diameter of the left atrium (DLA), the dimensions of the interventricular septum (IVS), posterior wall of the left ventricle (PWLV), the end diastolic diameter of the left ventricle (EDLV), the ejection fraction (EF), and the shortening fraction (SF).

All of the measurements were performed by the same certified physician.

### 4.7. Statistical Analysis

The numerical data was presented as mean ± standard deviation (SD) and the categorical data was presented as the frequency (%). Differences among groups were analyzed using ANOVA and Kruskal-Wallis tests. Correlations between groups or variables were found using Spearman’s correlation coefficient. All statistical analyses were performed using SPSS v.17 statistical software (v.17, SPSS Inc., Chicago, IL, USA). A *p*-value < 0.05 was considered statistically significant.

### 4.8. Compliance with the Ethical Standards

The present study has been approved by the Ethical Committee (29 June 2016) of the “Victor Babes” University of Medicine and Pharmacy, Timisoara, Romania, no. 7/2016. All procedures performed in this study with human participants were in accordance with the ethical standards of the institutional research committee and with the 1964 Helsinki declaration and its later amendments or comparable ethical standards.

## 5. Conclusions

The present study is innovative because, to our knowledge, no other study assessed the efficacy of Perindopril in hypertensive patients with endothelial dysfunction by quantifying TSP-1 plasma levels, compared with other antihypertensive drugs such as beta blockers, calcium channel blockers, and diuretics. Compared with other antihypertensive drugs that were studied here, Perindopril has better properties in reversing and controlling the processes of endothelial dysfunction. In the future, a study that quantifies the effects of an ARB (angiotensin receptor blocker) on TSP-1 plasma levels in hypertensive patients with ED will be necessary.

## Figures and Tables

**Table 1:** Characteristics of the control and study groups.

Parameters	Group A (*n* = 105)	Group B (*n* = 117)	Group C (*n* = 129)	*p*
Age (years)	55.13 ± 14.59	62.24 ± 13.54 (1)	61.24 ± 12.3 (1)	<0.001
Men	55 (105)	48 (117)	57 (129)	1
Women	50 (105)	69 (117)	72 (129)	-
HTN duration (months)	-	107.61 ± 86.47 (1)	104.00 ± 77.18 (1)	0.729
SBP (mmHg)	122.53 ± 17.81	139.58 ± 18.16 (1)	139.88 ± 16.39 (1)	<0.001
DBP (mmHg)	76.75 ± 9.95	83.69 ± 13.68 (1)	84.51 ± 10.76 (1)	<0.001
Heart rate (beats/min)	70.23 ± 8.46	76.19 ± 10.84 (2)	69.14 ± 7.18 (2)	<0.001

Data are represented as mean ± standard deviation (SD). HTN: hypertension, SBP: systolic blood pressure, DBP: diastolic blood pressure. Statistical significance was considered at a *p*-value < 0.05. (1) A significant difference when compared with the control group; (2) A significant difference between both hypertensive groups.

**Table 2:** Biochemical data among the patients in the study groups.

Parameters	Group A (*n* = 105)	Group B (*n* = 117)	Group C (*n* = 129)	*p*
ALAT (U/L)	39.19 ± 15.63	40.07 ± 15.49	42.58 ± 16.12	0.226
ASAT (U/L)	24.82 ± 9.16	23.97 ± 8.35	25.38 ± 10.65	0.509
Cholesterol (mg/dL)	198.10 ± 55.44	203.30 ± 51.75 (2)	216.47 ± 59.44 (2)	0.03
Triglicerides (mg/dL)	124.00 ± 72.6	148.21 ± 95.61	135.91 ± 57.62	0.347
Glucose (mg/dL)	110.16 ± 50.63	112.80 ± 45.73	109.69 ± 21.23	0.814
Creatinine (mg/dL)	0.85 ± 0.22	0.87 ± 0.20 (2)	0.96 ± 0.30 (2)	<0.001
Potassium (mmol/L)	4.36 ± 0.40	4.25 ± 0.46	4.38 ± 0.44	0.06
INR	1.16 ± 0.32	1.06 ± 0.25	1.29 ± 1.37	0.11
ESR (mm/h)	9.16 ± 5.47	13.21 ± 7.75 (2)	16.72 ± 13.79 (2)	<0.001
BUN (mg/dL)	18.43 ± 6.48	17.66 ± 7.04	18.84 ± 6.81	0.391

Data are represented as mean ± SD. Statistical significance was considered at a *p*-value < 0.05. (1) A significant difference when compared with the control group; (2) A significant difference between both hypertensive groups. ALAT: alanine aminotransferase (GTP). ASAT: aspartate aminotransferase (GOT). INR: international normalized ratio. ESR: erythrocyte sedimentation rate. BUN: blood urea nitrogen.

**Table 3:** The principal indicators of the presence/absence of ED.

Parameters	Group A (*n* = 105)	Group B (*n* = 117)	Group C (*n* = 129)	*p*
hs-CRP (mg/dL)	0.26 ± 0.35	0.26 ± 0.25	0.40 ± 0.56	0.007
Pentraxin-3 (ng/mL)	1.43 ± 1.35	1.35 ± 1.24	0.89 ± 0.58	<0.001
Thrombospondin-1 (ng/mL)	10882 ± 7290	10221 ± 6141.7	12117 ± 6895	0.08
FMD (%)	20 ± 0.3	10.63 ± 0.3	14 ± 0.03	<0.001
ED	absent	present	present	-
CC IMT right (mm)	0.83 ± 0.26	0.92 ± 0.19	0.95 ± 0.21	<0.001
CC IMT left (mm)	0.83 ± 0.21	0.95 ± 0.18	0.92 ± 0.18	<0.001
BMI (kg/m^2^)	23.94 ± 3.77	26.48 ± 4.00	28.06 ± 3.44	<0.001
Smokers	21 (105)	18 (117)	30 (129)	-

Data are represented as mean ± SD. Statistical significance was considered at a *p*-value < 0.05. hs-CRP: high-sensitivity C-reactive protein. PTX3: pentraxin-3. TSP-1: thrombospondin-1. FMD: flow mediated vasodilatation. ED: endothelial dysfunction. CC IMT: common carotid intima-media thickness. BMI: body mass index.

**Table 4:** Echocardiography data in different study groups.

Parameters	Group A (*n* = 105)	Group B (*n* = 117)	Group C (*n* = 129)	*p*
DLA (mm)	35.17 ± 6.88	37.61 ± 7.54	36.46 ± 5.70	0.02
IVS (mm)	10.76 ± 2.50	11.35 ± 2.26	11.25 ± 1.82	0.103
PWLV (mm)	10.04 ± 1.60	10.40 ± 1.85	10.88 ± 1.53	<0.001
EDLV (mm)	49.8 ± 6.79	52.56 ± 5.42	50.93 ± 4.82	0.001
E.F. (%)	56.01 ± 8.22	53.10 ± 8.65	55.46 ± 7.91	0.01
S.F. (%)	29.14 ± 5.80	28.10 ± 7.11	29.11 ± 5.01	0.322

Data are represented as mean ± SD. Statistical significance was considered at a *p*-value < 0.05. DLA: diameter of left atrium. IVS: diameter of interventricular septum. PWLV: posterior wall of the left ventricle. EDLV: end diastolic diameter of left ventricle. EF: ejection fraction. S.F.: shortening fraction.

**Table 5:** Correlation of TSP-1 plasma levels with all data in Group C.

Parameters	Thrombospondin-1	Correlation
Age (years)	----	*r* = 0.025, *p* < 0.001
HTN duration (months)	----	*r* = 0.022, *p* < 0.001
SBP (mmHg)	----	*r* = 0.005, *p* < 0.001
DBP (mmHg)	----	*r* = 0.045, *p* < 0.001
Cholesterol (mg/dL)	----	*r* = 0.0004, *p* < 0.001
Triglicerides (mg/dL)	----	*r* = 0.01, *p* < 0.001
Creatinine (mg/dL)	----	*r* = 0.001, *p* < 0.001
Glucose (mg/dL)	----	*r* = 0.015, *p* < 0.001
Potassium (mmol/L)	----	*r* = 0.005, *p* < 0.001
Heart rate (beats/min)	----	*r* = 0.009, *p* = 0.02
hs-CRP (mg/dL)	----	*r* = 0.015, *p* < 0.001
PTX3 (ng/mL)	----	*r* = 0.126, *p* < 0.001
FMD (%)	----	*r* = 0.012, *p* = 0.047
CC IMT right (mm)	----	*r* = 0.00001, *p* < 0.001
CC IMT left (mm)	----	*r* = 0.0002, *p* < 0.001
BMI (kg/m^2^)	----	*r* = 0.002, *p* < 0.001
WBC µL	----	*r* = 0.0002, *p* < 0.001
NEUTRO µL	----	*r* = 0.006, *p* < 0.001

----, no correlation.

**Table 6:** Correlation of TSP-1 plasma levels with all data in Group B.

Parameters	Thrombospondin-1	Correlation
Age (years)	----	*r* = 0.109, *p* < 0.001
HTN duration (months)	----	*r* = 0.019, *p* < 0.001
SBP (mmHg)	----	*r* = 0.00005, *p* = 0.018
DBP (mmHg)	----	*r* = 0.1069, *p* = 0.561
Cholesterol (mg/dL)	----	*r* = 0.028, *p* = 0.007
Triglicerides (mg/dL)	Positive	*r* = 0.329, *p* < 0.001
Creatinine (mg/dL)	----	*r* = 0.09, *p* = 0.271
Glucose (mg/dL)	----	*r* = 0.108, *p* < 0.001
Potassium (mmol/L)	----	*r* = 0.002, *p* = 0.167
Heart rate (beats/min)	----	*r* = 0.139, *p* = 0.131
hs-CRP (mg/dL)	----	*r* = 0.06, *p* < 0.001
PTX3 (ng/mL)	----	*r* = 0.101, *p* < 0.001
FMD (%)	----	*r* = 0.064, *p* = 0.863
CC IMT right (mm)	----	*r* = 0.036, *p* < 0.001
CC IMT left (mm)	----	*r* = 0.011, *p* < 0.001
BMI (kg/m^2^)	----	*r* = 0.086, *p* = 0.648
WBC µL	Positive	*r* = 0.334, *p* = 0.587
NEUTRO µL	Positive	*r* = 0.30, *p* = 0.0190

**Table 7:** Correlation of TSP-1 plasma levels with all data in Group A.

Parameters	Thrombospondin-1	Correlation
Triglicerides (mg/dL)	----	*r* = 0.0002, *p* < 0.001
WBC µL	----	*r* = 0.0003, *p* < 0.001
NEUTRO µL	----	*r* = 0.0002, *p* < 0.001

## Abbreviations

ACE inhibitors	angiotensin-converting enzyme inhibitors
Ang II	angiotensin II
BMI	body mass index
CC–IMT	common carotid intima-media thickness
DBP	diastolic blood pressure
DLA	diameter of the left atrium
ECM	extracellular matrix
ED	endothelial dysfunction
EDLV	end diastolic diameter of left ventricle
EDHF	endothelial derived hyperpolarizing factor
EF	ejection fraction
FMD	flow-mediated vasodilatation
HTN	hypertension
hs-CRP	high-sensitivity C-reactive protein
IVS	interventricular septum
MONO	monocytes
NEUTRO	neutrocytes, neutrophils
NO	nitric oxide
PAI	platelet activator inhibitor
PTX3	pentraxin-3
PWLV	posterior wall of the left ventricle
SD	standard deviation
SBP	systolic blood pressure
sGC	soluble guanylatecyclase
TGF	transforming growth factor
TSP-1	thrombospondin-1
VSMCs	vascular smooth muscle cells
WBC	white blood cells
